# Sensitivity Analysis of Ion Channel Conductance on Myocardial Electromechanical Delay: Computational Study

**DOI:** 10.3389/fphys.2021.697693

**Published:** 2021-08-27

**Authors:** Ali Ikhsanul Qauli, Aroli Marcellinus, Ki Moo Lim

**Affiliations:** Department of IT Convergence Engineering, Kumoh National Institute of Technology, Gumi, South Korea

**Keywords:** myocardial action potential, action potential duration, electromechanical delay, cardiac arrhythmia, computational simulation

## Abstract

It is well known that cardiac electromechanical delay (EMD) can cause dyssynchronous heart failure (DHF), a prominent cardiovascular disease (CVD). This work computationally assesses the conductance variation of every ion channel on the cardiac cell to give rise to EMD prolongation. The electrical and mechanical models of human ventricular tissue were simulated, using a population approach with four conductance reductions for each ion channel. Then, EMD was calculated by determining the difference between the onset of action potential and the start of cell shortening. Finally, EMD data were put into the optimized conductance dimensional stacking to show which ion channel has the most influence in elongating the EMD. We found that major ion channels, such as L-type calcium (CaL), slow-delayed rectifier potassium (Ks), rapid-delayed rectifier potassium (Kr), and inward rectifier potassium (K1), can significantly extend the action potential duration (APD) up to 580 ms. Additionally, the maximum intracellular calcium (Cai) concentration is greatly affected by the reduction in channel CaL, Ks, background calcium, and Kr. However, among the aforementioned major ion channels, only the CaL channel can play a superior role in prolonging the EMD up to 83 ms. Furthermore, ventricular cells with long EMD have been shown to inherit insignificant mechanical response (in terms of how strong the tension can grow and how far length shortening can go) compared with that in normal cells. In conclusion, despite all variations in every ion channel conductance, only the CaL channel can play a significant role in extending EMD. In addition, cardiac cells with long EMD tend to have inferior mechanical responses due to a lack of Cai compared with normal conditions, which are highly likely to result in a compromised pump function of the heart.

## Introduction

In the modern world, cardiovascular disease (CVD) remains a significant health burden worldwide (Virani et al., 2020). One prominent example of CVD is dyssynchronous heart failure (DHF), which is characterized by compromised pump function. In common DHF, the workload is typically highest in the left ventricle and lowest in the septum and is accompanied by regional differences in wall stress (Vernooy et al., 2007). This condition leads to regional differences in wall thickness and chamber remodeling (Gao et al., 2008). One key factor in assessing DHF is electromechanical delay (EMD), which describes the time difference between the onset of action potential and starting time of myofiber shortening during the excitation-contraction process (Cordeiro et al., 2004). Electromechanical delay has two components: (a) the intrinsic latent period between the depolarization and myofilament (MF) activation in myocytes (Cordeiro et al., 2004) and (b) local myofiber mechanical loading conditions in an intact heart (Russell et al., 2011).

Furthermore, several studies have reported some experimental techniques for measuring EMD and its clinical implications in cardiac muscle, particularly in patients with DHF. Frank et al. recently have reported novel systolic stretch and diastolic relaxation discoordination indexes derived from cardiac magnetic resonance imaging to investigate the clinical and mechanistic implications of left ventricular (LV) electromechanical dyssynchrony in children with pulmonary arterial hypertension (Frank et al., 2020). They revealed that there is evidence of right ventricular-induced LV discoordination, including a combination of delayed early systolic electromechanical activation, late systolic septal shift, and prolonged postsystolic septal thickening. A recent report by Li et al. (2019) emphasizes the clinical importance of EMD, in which EMD variation can improve the detection of coronary atrial disease. Another variant of EMD, the atrial EMD, is well-known for predicting atrial fibrillation (Bennett, 1984), and tissue Doppler imaging (TDI) has also revealed that atrial EMD is significantly associated with stroke (Akil et al., 2015). In addition to various experimental methods for detecting EMD at the muscular level, such as TDI, there is an experimental approach for measuring EMD at the single-cell level, namely, light-sheet fluorescence microscopy (LSFM), as reported by Turaga et al. (2020). They revealed that LSFM could be utilized to determine structure-function relationships both at the tissue level and at single-cell resolution.

Despite its clinical importance and findings in treating patients with DHF, experimental studies of EMD are constrained by the limitations of experimental apparatus to monitor and access numerous molecular dynamics of ventricular tissue in generating EMD and other cellular activities. Therefore, researchers have been developing electrical and mechanical models of cardiac cells to enable further exploration that experimental studies cannot accomplish. The computational study of EMD relies on two aspects of simulation: the electrical and mechanical activities of cardiac cells. Various models of myocyte cells are currently available, and most are inspired by the work of Hodgkin and Huxley on cellular excitability (Hodgkin and Huxley, 1952). A recent model proposed by ten Tusscher and Panfilov incorporates sarcoplasmic reticulum (SR) calcium dynamics through the Markov model to trigger mechanical cross-bridges in the cell (ten Tusscher and Panfilov, 2006). In addition, another model proposed by O'Hara et al. (2011) has similar features, although it has fewer transmembrane ion channels than the one proposed by ten Tusscher and Panfilov. As for the mechanical cross-bridge cycle of the common muscle tissue, Eisenberg and Greene (1980) proposed a model that can simulate the transition from weak binding to strong binding and back to weak binding while releasing a positive force. In addition, Rice et al. (2008) proposed a lumped model of the cardiac MF that allows the simulation of the mechanical contraction of the isolated cell. Therefore, as the mathematical model for electrical and mechanical simulation of cardiac cells is readily available, a computational study can reveal the detailed results of EMD and its clinical consequences.

Recent computational studies by Gurev et al. (2010) have described that the EMD also depends on the mechanical loading condition of the intact heart, and its distribution is clearly different during sinus rhythm and epicardial pacing. In addition, a study by Constantino et al. (2012) revealed that the optimal cardiac resynchronization therapy (CRT) strategy in the DHF heart could be achieved by pacing at the LV location with the longest EMD. In addition, some recent studies have revealed that computational studies can predict the total reduction of EMD for CRT (with or without an LV assist device) on the right bundle branch block and left bundle branch block heart (Heikhmakhtiar and Lim, 2018) and the assessment of EMD during sinus rhythm, tachycardia, and ventricular fibrillation conditions (Heikhmakhtiar and Lim, 2020). However, most studies focus on the 3D perspective of the heart; hence, there is no clear description of which channels affect EMD the most during the simulation and the role of the interplay of ion channel conductivity of the cardiac cell in the prolongation of EMD.

In this work, we studied the cellular dynamics of EMD and the sensitivity of each ion channel in prolonging EMD through the variation in the maximum conductance. By doing so, we mimicked the electrical mechanical remodeling of the heart wall, which influences the conductance of the ion channel.

## Methods

Here, we reviewed the model of myocardial cells based on the study of ten Tusscher and Panfilov (2006), and then we briefly explained the model for crossbridge mechanisms based on the study of Rice et al. (2008). Finally, we described the simulation protocol for obtaining the EMD.

### The Electrical Model of Human Ventricular Tissue

**Figure 1:**
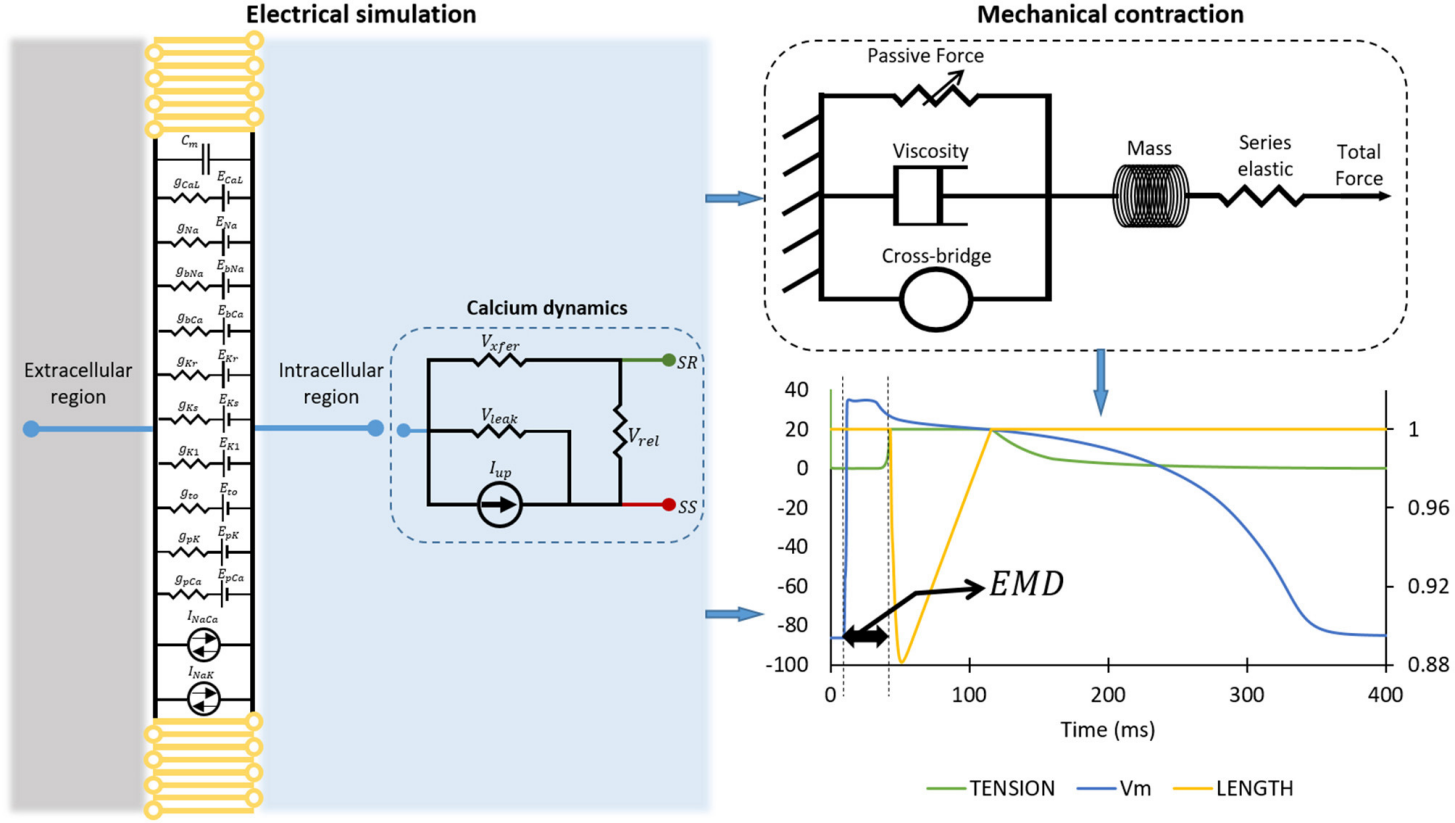
The simulation protocol for obtaining two EMDs. The diagram on the left panel shows the mode of myocardial tissue. The mechanical contraction of the cell is depicted on the top right of the figure, while the bottom right of the figure shows how we calculated EMD from the electrical action potential and mechanical contraction. Please note that the plot on the bottom right has the primary *x*-axis as time in milliseconds, the *y*-axis as membrane potential of the cell in millivolt, and the secondary *y*-axis (on the right) as the normalized length of the cell.

The ventricular model incorporated in this work is the developed version of the model proposed by ten Tusscher et al. (2004), which is based on restitution data (Nash et al., 2006). The cardiac myocyte model proposed by ten Tusscher and Panfilov (2006) includes a more comprehensive description of calcium dynamics, subsarcolemmal space, and calcium-induced calcium release (CICR). This model of calcium dynamics can be an excellent instrument to study the excitation and contraction coupling of cardiac cells. A schematic diagram of the ventricular cell model is shown in the left panel of Figure 1. Ten ion channels incorporated in the model allow transmembrane ionic currents to flow either inward or outward the cell. The membrane potential (*V*) of a cell can be described as follows:

$$\begin{array}{l} C_{m}\frac{dV}{dt}=-\left(I_{ion}+I_{stim}\right) \end{array}$$

where *C*_*m*_ is the membrane capacitance, *I*_*stim*_ is the stimulus current, and *I*_*ion*_ is the sum of the ionic transmembrane currents. The corresponding ionic currents are sodium current (*I*_*Na*_), inward rectifier potassium current (*I*_*K*1_), transient outward current (*I*_*to*_), rapid-delayed rectifier potassium current (*I*_*Kr*_), slow-delayed rectifier potassium current (*I*_*Ks*_), L-type calcium current (*I*_*CaL*_), plateau calcium current (*I*_*pCa*_), plateau potassium current (*I*_*pK*_), background calcium current (*I*_*bCa*_), background sodium current (*I*_*bNa*_), sodium-calcium exchanger current (*I*_*NaCa*_), and sodium-potassium pump current (*I*_*NaK*_). In addition to the transmembrane ionic current, there is an intracellular current called calcium dynamics, which is represented by the Markov model with a four-state ryanodine receptor based on the one proposed by Shannon et al. (2004) and Stern et al. (1999). There are four available states for the Markov model: (i) *O* is the open-conducting state, (ii) *R* is the resting closed state, (iii) *I* is the inactivated closed state, and (iv) *RI* is the resting inactivated closed state. The *k*_3_ and *k*_4_values are constant, while *k*_1_ and *k*_2_ depend on the SR calcium concentration. To reduce the computational load, the following set of equations is used to describe the Markov model of the ryanodine receptor:

$$\begin{array}{l} \frac{d\bar{R}}{dt}=-k_{2}Ca_{ss}\bar{R}+k_{4}\left(1-\bar{R}\right)\\ O=\frac{k_{1}Ca_{ss}^{2}\bar{R}}{k_{3}+k_{1}Ca_{ss}^{2}}\\ I_{rel}=V_{rel}O\left(Ca_{SR}-Ca_{ss}\right) \end{array}$$

where $\bar{R}=R+O$, *Ca*_*SR*_ is the free SR Ca^2+^ concentration, *Ca*_*SS*_ is the free diadic subspace Ca^2+^ concentration, *I*_*rel*_ is the CICR current, and *V*_*rel*_ is the maximal *I*_*rel*_ conductance.

Because we will focus on excitation and contraction coupling of the cardiac cell, the detailed description of the dynamic of cytoplasmic or intracellular calcium (Cai) concentration will be very important. Aligned with the schematic description of calcium dynamic in the left panel of Figure 1, the *I*_*CaL*_ enters the subspace (SS) and induces the release of calcium from SR to SS through the *I*_*rel*_. After that, the diffusion of calcium from SS to the cytoplasmic region is carried out by *I*_*xfer*_. The *I*_*NaCa*_ pumps out the calcium from cytoplasm to the exterior region of cell. Another calcium pump current *I*_*up*_ pushes the calcium back to the SR, while the leak current *I*_*leak*_ leaks calcium from SR to cytoplasm. Therefore, the mathematical formalism for Cai concentration is as follows:

$$\begin{array}{l} \frac{dCai_{total}}{dt}=-\frac{I_{bCa}+I_{pCa}-2I_{NaCa}}{2V_{c}F}+\frac{V_{SR}}{V_{c}}\left(I_{leak}-I_{up}\right)+I_{xfer},\\ \frac{dCa_{SRtotal}}{dt}=I_{up}-I_{leak}-I_{rel},\\ \frac{dCa_{SStotal}}{dt}=-\frac{I_{CaL}}{2V_{SS}F}+\frac{V_{SR}}{V_{SS}}I_{rel}-\frac{V_{C}}{V_{SS}}I_{xfer}, \end{array}$$

where *V*_*SR*_ is the SR volume of 1.094 μm^3^, *V*_*SS*_ is the subspace volume of 0.05468 μm^3^, and *F* is Faraday constant of 96.4867 C/mmol. A more complete description of the formalism for each ionic current as well as the calcium dynamics can be found in ten Tusscher and Panfilov (2006) and references therein.

### The Mechanical Model of the Cell

The model of the mechanical contraction of the cell is depicted in the top-right panel of Figure 1. As the preliminary procedure for mechanical contraction of the cell through crossbridge formations, the binding of calcium and troponin can be explained by introducing two troponin population sites that correspond to high (*CaTrop*_*H*_) and low (*CaTrop*_*L*_) affinity sites that are expressed as follows:

$$\begin{array}{l} \frac{d}{dt}CaTrop_{H}=k_{onT}{\left[Ca\right]}_{i}\left(1-CaTrop_{H}\right)-k_{offHT}CaTrop_{H},\\ \frac{d}{dt}CaTrop_{L}=k_{onT}{\left[Ca\right]}_{i}\left(1-CaTrop_{L}\right)-k_{offLT}CaTrop_{L}, \end{array}$$

where *k*_*onT*_ is the complete rate constant for binding, [*Ca*]_*i*_ is the calcium concentration (taken from total Cai concentration in the electrical simulation of the cell), *k*_*offHT*_ is the complete rate constant to unbind from high-affinity sites, and *k*_*offLT*_ is the complete rate constant to unbind the low-affinity sites. Furthermore, the calcium activation on the interaction of troponin and tropomyosin is modeled with a system of ordinary differential equations (ODE). To model the calcium activation, troponin and tropomyosin are assumed as regulatory units that inherit one of the two states, the non-permissive state (N) and the permissive state (P). The N state prevents crossbridge formation states, while, in contrast, the P state permits the transition to strongly bound crossbridge conditions. In the case there is no crossbridge formation exists, the states of troponin and tropomyosin can be described as follows:

$$\begin{array}{l} \frac{d}{dt}N_{NoXB}=-k_{n-pT}\times N_{NoXB}+k_{p-nT}\times P_{NoXB},\\ \frac{d}{dt}P_{NoXB}=k_{n-pT}\times N_{NoXB}-k_{p-nT}\times P_{NoXB}, \end{array}$$

where *k*_*n*−*pT*_ and *k*_*p*−*nT*_ are the transition rates. These transition rates have non-linear formulation as follows:

$$\begin{array}{l} Trop_{Regulatory}\left(x\right)=\left(1-SOVF_{thin}\left(x\right)\right)\times CaTrop_{L}\\ +SOVF_{thin}\left(x\right)\times CaTrop_{H}, \end{array}$$

where the fraction of Ca-bounded thin filament regulatory units is represented by *Trop*_*Regulatory*_(*x*), the sarcomere length is *x*, and, finally, *SOVF*_*thin*_(*x*) is the single-overlap function of the thin filament.

Furthermore, the function representing the change of the regulatory unit to a permissive state is expressed as follows:

$$\begin{array}{l} permtot={\left(\frac{1}{\left(1+{\left(\frac{perm_{50}}{Trop_{Regulatory}\left(x\right)}\right)}^{n_{perm}}\right)}\right)}^{0.5}, \end{array}$$

where *perm*_50_ = 0.5 is the half-activation constant and *n*_*perm*_ = 15 is the Hill coefficient. The *permtot* function can alter the *k*_*n*−*pT*_ rate as follows:

$$\begin{array}{l} k_{n-pT}=k_{n-p}\times permtot\times Qk_{n-p}^{\left(\frac{\left(TmpC-37\right)}{10}\right)}, \end{array}$$

where *k*_*n*−*p*_ = 50 s^−1^, *Qk*_*n*−*p*_ = 1.6, and *TmpC* is the temperature in degree Celsius. Inversely, for the transition rate from the permissive to the non-permissive state, the inverse function of *permtot* is expressed as follows:

$$\begin{array}{l} inverse\;permtot=\min\left(\frac{1}{permtot},100\right). \end{array}$$

Therefore, the *k*_*p*−*nT*_ transition rate is:

$$\begin{array}{l} k_{p-nT}=k_{p-n}\times inversepermtot\times Qk_{p-n}^{\left(TmpC-37\right)}, \end{array}$$

where *k*_*p*−*n*_ = 500 s^−1^, *Qk*_*p*−*n*_ = 1.6.

Moreover, in the case where crossbridge formations exist, state occupancy of the non-permissive state (*N*_*XB*_) and the permissive state (*P*_*XB*_), together with the strongly bound state prior to isomerization (*XB*_*PreR*_) and the post-isomerization state (*XB*_*PostR*_) can be expressed as follows:

$$\begin{array}{l} \frac{d}{dt}N_{XB}=-k_{n-pT}\times N_{XB}+k_{p-nT}\times P_{XB},\\ \frac{d}{dt}P_{XB}=k_{n-pT}\times N_{XB}-\left(k_{p-nT}+f_{appT}\right)\\ \times P_{XB}+g_{appT}\times XB_{PreR}+g_{xbT}\times XB_{PostR},\\ \frac{d}{dt}XB_{PreR}=f_{appT}\times P_{XB}-\left(g_{appT}+h_{fT}\right)\times XB_{PreR}\\ +h_{bT}\times XB_{PostR},\\ \frac{d}{dt}XB_{PostR}=h_{fT}\times XB_{PreR}-\left(h_{bT}+g_{xbT}\right)\times XB_{PostR}, \end{array}$$

where *f*_*appT*_, *g*_*appT*_, *g*_*xbT*_, *h*_*fT*_, and *h*_*bT*_ are the transition rates. Moreover, as proposed by Razumova et al. (1999), the force or tension generated from crossbridge formation depends on the multiplication of strongly bound states (*XB*_*PreR*_ and *XB*_*PostR*_) and its average distortion (*xXB*_*PreR*_ and *xXB*_*PostR*_). The mean distortion of strongly bound states can be expressed as follows:

$$\begin{array}{l} \frac{d}{dt}xXB_{PreR}=\frac{1}{2}\frac{dSL}{dt}+\frac{\phi }{XB_{PreR}^{DutyFract}}\times \\ \;[f_{appT}\times \left(-xXB_{PreR}\right)+h_{bT}\\ \;\times \left(xXB_{PostR}-x_{0}-xXB_{PreR}\right)],\\ \frac{d}{dt}xXB_{PostR}=\frac{1}{2}\frac{dSL}{dt}+\frac{\phi }{XB_{PostR}^{DutyFract}}\\ \;\left[h_{fT}\times \left(xXB_{PreR}+x_{0}-xXB_{PostR}\right)\right],\\ XB_{PreR}^{DutyFract}=\frac{f_{appT}h_{bT}+f_{appT}g_{xbT}}{\begin{array}{l} g_{xbT}h_{fT}+f_{appT}h_{fT}+g_{appT}h_{bT}+g_{appT}g_{xbT}\\ \;+f_{appT}h_{bT}+f_{appT}g_{xbT} \end{array}},\\ XB_{PostR}^{DutyFract}=\frac{f_{appT}h_{fT}}{\begin{array}{l} g_{xbT}h_{fT}+f_{appT}h_{fT}+g_{appT}h_{bT}+g_{appT}g_{xbT}\\ \;+f_{appT}h_{bT}+f_{appT}g_{xbT} \end{array}}, \end{array}$$

where (*dSL*/*dt*) represents how quick the sarcomere length change is, ϕ is the scaling factor that is determined empirically, and *x*_0_ is the mean strain of the strongly bound state.

Given the state occupancy for crossbridge formation, we can obtain the normalized active force under optimal condition, where *k*_*n*−*pT*_ ≫ *k*_*p*−*nT*_ as follows:

$$\begin{array}{l} F_{active}\left(x\right)=SOVF_{thick}\left(x\right)\\ \times \frac{xXB_{PreR}\times XB_{PreR}+xXB_{PostR}\times XB_{PostR}}{x_{0}\times XB_{PostR}^{Max}}, \end{array}$$

where *SOVF*_*thick*_(*x*) is the scaling term representing the effect of sarcomere geometry to the crossbridge formation, and *x* is the sarcomere length. Other forces participating in the generation of total force are passive force (*F*_*passive*_(*x*)), constant preload force (*F*_*preload*_ = *F*_*passive*_(*SL*_0_)) that will trigger sarcomere length larger than its resting length, and, lastly, the afterload force (*F*_*afterload*_(*x*)), which is constant during isotonic contraction while, in isometric (fixed length) contraction, it represents as series of elastic elements, as shown in top-right-panel Figure 1, the form of:

$$\begin{array}{l} F_{afterload}\left(x\right)=KSE\times \left(x-SL_{0}\right), \end{array}$$

where *KSE* is the stiffness in units of normalized force per μm, and *SL*_0_ is the initial value of *SL* (the sarcomere length). Finally, the complete muscle simulation can be calculated through some changes in sarcomere length caused by forces as follows:

$$\begin{array}{l} \frac{d}{dt}SL\;=\;\frac{Integral_{Force}+\left(SL_{0}-SL\right)\times viscosity}{mass} \end{array}$$

where the *viscosity* factor is set as the muscle is assumed to have a Newtonian viscosity, *mass* is added to prevent instantaneous changes in muscle-shortening velocity, and *Integral*_*Force*_ is the integrated normalized force in the form of the following equation:

$$\begin{array}{l} Integral_{Force}=\int_{0}^{t}(F_{active}\left(x\right)+F_{passive}\left(x\right)-F_{preload}\\ \;-F_{afterload}\left(x\right))dt. \end{array}$$

A more detailed explanation of the corresponding formulas and equations can be found in the Rice et al. (2008) and references included therein.

### Simulation Protocol

The computational simulation to obtain the EMD consists of several steps. First, we ran an electrical simulation of cardiac cells by varying the ion channel conductance. As reported by Rahm et al. (2018), most of the heart failure may undergo ion channel remodeling such as change in ion channel function, composition, and localization. It can also include histological modifications such as fibrosis. Specifically, for ion channels remodeling, some studies reported remodeling in ionic current in patients with heart failure; the decrease of *I*_*to*_ current (Beuckelmann et al., 1993; Kaab et al., 1996; Rozanski et al., 1997; Li et al., 2000, 2002; Pogwizd et al., 2001; Zicha et al., 2004; Rose et al., 2005; Tsuji et al., 2006), the decrease of *I*_*Ks*_ current (Li et al., 2000, 2002; Tsuji et al., 2000, 2006), the decrease of *I*_*Kr*_ current (Tsuji et al., 2000, 2006), no change in *I*_*Kr*_ current (Li et al., 2000, 2002), decrease of *I*_*K*1_ current (Beuckelmann et al., 1993; Kaab et al., 1996; Li et al., 2000, 2002; Pogwizd et al., 2001; Rose et al., 2005), no change in *I*_*K*1_ current (Rozanski et al., 1997; Tsuji et al., 2000), and the decrease of *I*_*CaL*_ current (Ouadid et al., 1995; Mukherjee et al., 1998; Li et al., 2000). Furthermore, some studies reported that the genetic disorder of ion channels can also alter the function ion channels; long-QT-syndrome caused by reduction of *I*_*Kr*_ current (mutation of gene KCNQ1 and KCNH2) and increment of *I*_*Na*_ current (mutation of gene SCN5A) as reported by Ackerman et al. (2011) and Schwartz et al. (2012), Bugrada syndrome by the reduction of *I*_*Na*_ current (mutation of gene SCN5A, GPD1L, and SCN1B) as reported by Brugada and Brugada (1992) and reduction of *I*_*CaL*_ current by mutation of gene CACNA1C and CACNB2B as reported by Antzelevitch et al. (2007), and short-QT-syndrome caused by the increase of *I*_*Kr*_ current (mutation KCNQ1 and KCNH2) as reported by Bellocq et al. (2004) and Brugada et al. (2004), increase of *I*_*K*1_ current by mutation of gene KCNJ2 as reported by Priori et al. (2005). Moreover, the recent study by Mirams et al. (2011) and others under comprehensive *in vitro* proarrhthmiaassasy (CiPA) such as Li et al. (2016), Chang et al. (2017), and Dutta et al. (2017) examined the *torsade de pointes* risk of the drug that causes multiple ion channel blocking effects. Therefore, in this work, we focus on the reduction of the ion channel conduction to mimic a broad variety of ion channel blocking from previous studies. We deployed four stages of reduction by varying the conductance of each ion channel to 25, 50, 75, and 100% of the corresponding maximum conductance on all 10 ion channels available in the cell. This means that we had 4^10^ or 1,048,576 cases to run. In each case, we used two different basic cycle lengths (BCLs) as proposed in S1-S2 protocol by ten Tusscher et al. (2004) and ten Tusscher and Panfilov (2006), that is, 1,000 and 600 ms with 50 pacings in each case to ensure the simulation reached a proper steady state. A schematic diagram of the electrical simulation of the cell is shown in the left panel of Figure 1.

Results of the electrical cell simulation are the time series data of intracellular calcium (Cai) concentration and membrane potential that are taken from the last beat of the simulation. In addition, the action potential duration (APD) of the last beat is calculated based on the time taken for 90% repolarization. The time series of membrane potential and Cai act as input for mechanical cell simulation. Then, the mechanical simulation results in tension as well as normalized cell length as a function of time. Once the electrical and mechanical simulations of the cell are performed, the EMD can be calculated by determining the time difference between the firing of action potential with respect to the shortening of the cell.

The onset of the action potential is detected, and its time is captured when the membrane potential of the cell reach value is higher than or equal to −40 mV. Furthermore, for the length shortening, we assumed the cell undergoes contraction when the normalized length reduces to be ≤99% of its original size, and we recorded the time when the cell reached this condition. Finally, we calculated the EMD by subtracting the time at the start of the contraction of the cell with the time at the onset of the action potential. Furthermore, for the calcium attributes other than max Cai concentration, the resting Cai concentration is obtained from the last time series data of Cai concentration; the Cai duration is the time interval when the Cai is 90% from the peak, and, finally, Cai slope is the average gradient of 90% of the Cai peak to the time duration for Cai reaches its peak.

To visualize the conductance with the most effects, we followed dimensional stacking (Taylor et al., 2006) and its optimization as proposed by Gemmell et al. (2014). The optimization of dimensional stacking is performed by finding the minimum absolute difference between each point and its four neighbors in the *x*- and *y*-axes. This can smoothen the map as the “lower-order” conductance with a smaller effect on the APD, EMD, or calcium-transient characteristics variation is given the shorter line label. Therefore, the conductance with the highest effect will have the longest label on either the *x*- or *y*-axis. The number of available configurations is $\frac{10!}{2}$ because the interchangeable configuration between the *x*- and *y*-axes can result in the same absolute difference value. The axis configuration that results in the smallest summation of the absolute difference for the entire map is categorized as the optimum map. We will assess three quantities, that is, APD, max Cai, and EMD, to comprehensively examine ion channels affecting EMD prolongation.

## Results

**Figure 2:**
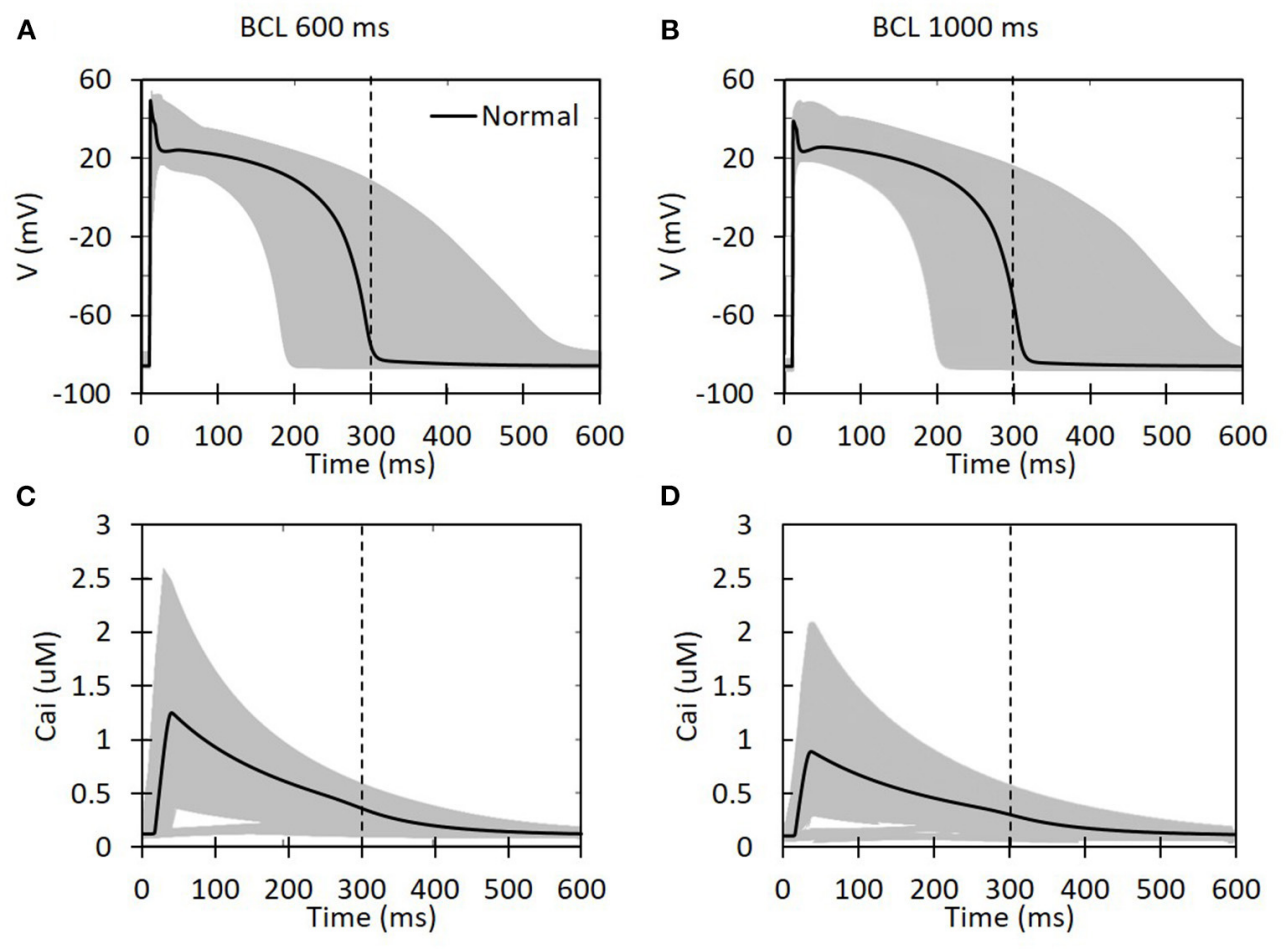
The action potential shape and intracellular calcium transient for all possible ion channel maximum conductance variations. The x-axis is time in milliseconds for all the panels. For **(A,B)**, the y-axis is the membrane potential in millivolt, and, for **(C,D)**, it is the intracellular calcium concentration in micromolar. The black solid line represents a case with normal maximum conductance for each ion channel (set to its 100% value). The vertical dashed line indicates 300 ms.

Based on the reduction of the maximum conductance for each ion channel as well as the BCL, the shape of the action potential for all available cases is presented in Figure 2. We can clearly observe that the decrease in the maximum conductance affects both the depolarization and repolarization of the action potential. During depolarization, some cases can yield a high membrane potential of up to 60 mV. Meanwhile, in the repolarization part, some cases can make a considerably longer plateau than the others. Basic cycle length variation also seems to affect APD. One indicator for this is that the normal case for the BCL 1,000 ms (Figure 2B) has slightly longer APD than the BCL 600 ms (Figure 2A) (the action potential line that crosses the 300-ms vertical dashed line). In addition, the shape of Cai concentration is also available in Figure 2 that shows a lower peak of calcium for BCL 1,000 ms compared with BCL 600 ms cases. Furthermore, the effects of BCL variation on Cai transient can be seen from Figures 2C,D. As we can see, for the normal case, the BCL 600 ms can generate max Cai of 1.247 μM that is higher than its BCL 1,000 ms counterpart with 0.889 μM. Similarly, the highest max Cai for BCL 600 ms is over 3 μM, and the one for BCL 1,000 ms is below 3 μM. In both BCL variations, we can observe that some conductance variation cases produce quite low Cai indicated by some lines at the bottom of Figures 2C,D that do not show a considerable jump of Cai concentration. Finally, we can also see that some cases delay the onset of Cai up to around 50 ms.

**Figure 3:**
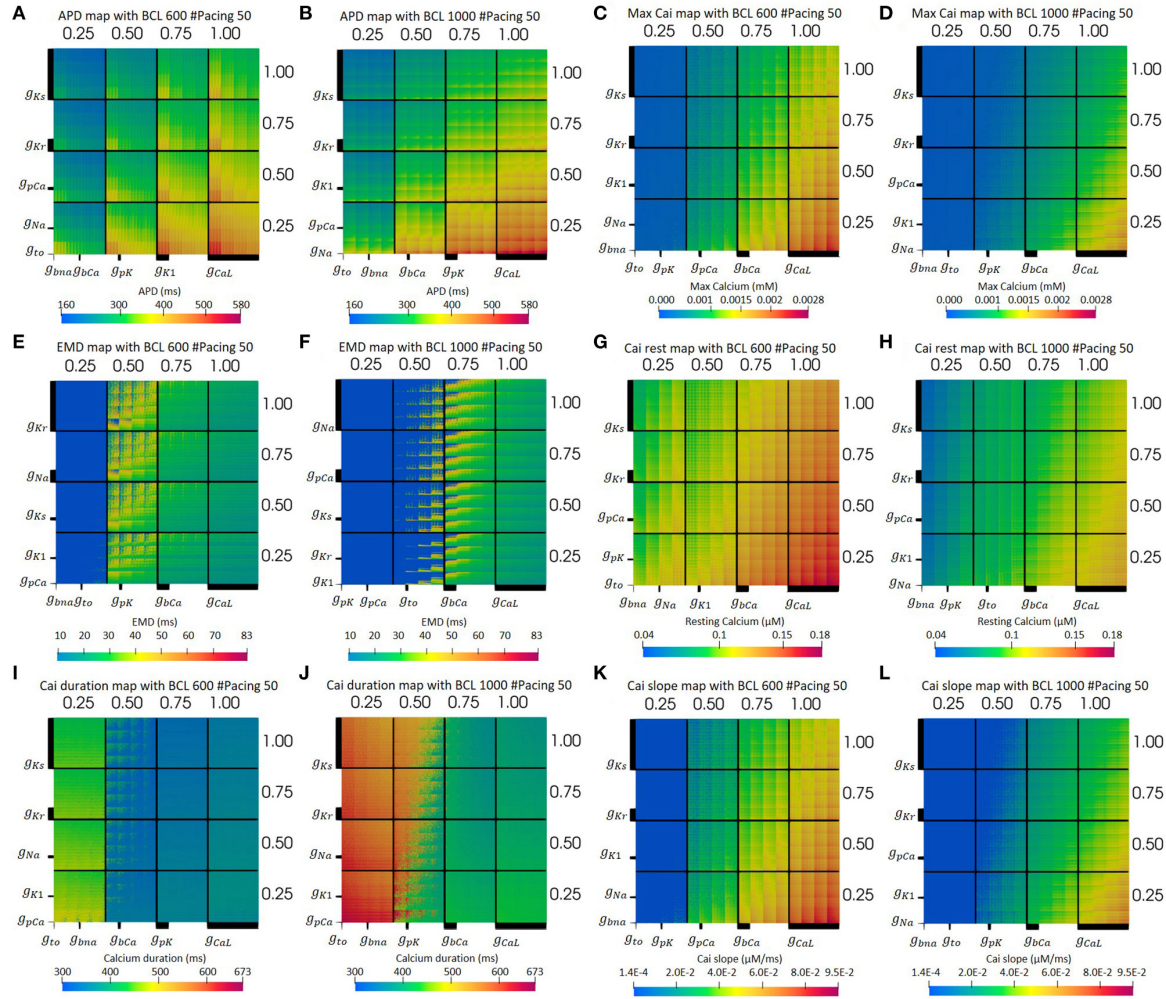
The optimum dimensional stacking for both BCL 600 ms **(A,C,E,G,I,K)** and BCL 1,000 ms **(B,D,F,H,J,L)**. Note that the bold line on each axis indicates the scale of each conductance variation. The biggest axis scale is 1/4 of the main axis, such as the CaL channel. The smaller one will have 1/4 the size of the bigger axis next to it. For **(E,F)**, the bold blue dots on the left part of the map are for no-contraction cases.

Figure 3 presents the electrical simulation results, involving 4^10^ cases. We deployed dimensional stacking, as proposed by Taylor et al. (2006) and Gemmell et al. (2014), which allows us to store multidimensional information in a two-dimensional map. From Figures 3A,B, we can clearly observe that the CaL and Ks channels significantly influence the overall prolongation of APD of the cell. At the level of 75–100% maximum CaL conductance and 25–50% of Ks conductance, the APD map has a yellow to red color, meaning the APD can reach up to a maximum value of 580 ms. In addition, from Figure 3A, we can find that, for BCL 600 ms, the K1 channel also influences APD prolongation, where the level of 25% K1 conductance yields long APD. Meanwhile, for BCL 1,000 ms in Figure 3B, the low level (25%) of pK channel variation also extends APD even though its effect is not as clear as the downgrading of K1 channel in BCL 600 ms.

Moreover, from the optimum max Cai map in Figures 3C,D, we can observe that the max Cai map is in line with the APD map in Figures 3A,B where the reduction in the maximum conductance of CaL and Ks channels significantly influences the max Cai concentration. The effect of bCa channel variation can also be seen quite clearly where a moderate to normal variation (50–100%) can generate high max Cai (yellow to red color) with a concentration of 0.0028 mM. For BCL 1,000 ms, the effect of bCa decrement can mostly be seen on the normal CaL channel, while, for BCL 600 ms, it is from the 75–100% level of the CaL channel. In addition, the downgrading of the Kr channel shows some influence in increasing the max Cai where its low to moderate (25–75%) variation can yield high max Cai. Finally, for the overall view of the map, BCL 600 ms shows more cases of high max Cai compared with that in BCL 1,000 ms.

The optimum EMD map can be seen from Figures 3E,F. Both BCL 1,000 and 600 ms showed no mechanical contraction on the left side of the Figures 3E,F (bold blue region). Meanwhile, the light blue region on the right side represents the short EMDs. From Figures 3E,F, it is clear that the long EMD (yellow to red color) appears mostly within the moderate condition (50–75%) of the CaL channel with maximum EMD detected to be 83 ms. For BCL 1,000 ms, long EMDs can be found mostly within the 50 and 75% variation of the CaL channel, whereas, for BCL 600 ms, it is within 50% variation. In addition, the low conductance variation (25%) of the CaL channel triggers no contraction cases indicated by bold blue dots grouping in this region. Furthermore, the reduction of the bCa channel also influences the EMD prolongation such that no-contraction cases appear more frequently as bCa decreases in conductance under the moderate condition of the CaL channel.

Other Cai transient characteristics are also available on Figure 3. Resting Cai concentration is depicted on Figures 3G,H. As we can see, most influential ion channels are CaL and Ks channels, followed by bCa and Kr channels. The axis configuration for both BCL variation of the map of resting Cai is the same as the one for the map of max Cai. However, the BCL 600 ms variation produces more cases with high-resting Cai concentration (yellow to red color) compared with its BCL 1,000 ms counterparts. Furthermore, the map of overall duration of Cai transient can be observed on Figures 3I,J. As we can see, the CaL and Ks channels influence the most on prolonging the Cai transient duration. Particularly, the (25–50%) level of CaL channel conductance can yield a quite distinct variation of Cai duration where there are some jumps from dark blue (short duration around 300 ms) to the yellowish green (duration of about 400–500 ms) region at BCL 600 ms, and there are transitions from dark green (approximately 400 ms) to reddish orange color (duration of approximately 600 ms) at BCL 1,000 ms. Finally, the slope of Cai transient is depicted on Figures 3K,L. From Figures 3K,L, the CaL and Ks channels affect the alteration of the Cai slope the most, followed by bCa and Kr channels. On Figure 3K, the BCL 600 ms condition, generally, can produce more cases with a high-Cai slope, depicted by the more yellow to red region (a slope of approximately 0.006–0.009 μM/ms) overall compared with the one in Figure 3L.

Furthermore, the summary of the axis configurations for the optimum map with the lowest absolute error is listed in Table 1. As we can see, each BCL has a different axis configuration for obtaining an optimum map. However, we can observe some patterns, especially for the most influential ion channels for prolonging the APD, increasing max Cai and, finally, extending EMD.

**Table 1:** The list of axis configuration for the optimum map.

**Biomarkers**	**Optimum configuration for BCL 600 ms**		**Optimum configuration for BCL 1,000 ms**	
	**x**	**y**	**x**	**y**
APD	bNa, bCa, pK, K1, CaL	to, Na, pCa, Kr, Ks	to, bNa, bCa, pK, CaL	Na, pCa, K1, Kr, Ks
Max Cai	to, pK, pCa, bCa, CaL	bNa, Na, K1, Kr, Ks	bNa, to, pK, bCa, CaL	Na, K1, pCa, Kr, Ks
EMD	bNa, to,pK, bCa, CaL	pCa, K1, Ks, Na, Kr	bNa, to, pK, bCa, CaL	K1, Kr, Ks, pCa, Na
Cai rest	bna, Na, K1, bCa, CaL	to, pK, pCa, Kr, Ks	bna, pK, to, bCa, CaL	Na, K1, pCa, Kr, Ks
Cai duration	to, bna, bCa, pK, CaL	pCa, K1, Na, Kr, Ks	to, bna, pK, bCa, CaL	pCa, K1, Na, Kr, Ks
Cai slope	to, pK, pCa, bCa, CaL	bna, Na, K1, Kr, Ks	bna, to, pK, bCa, CaL	Na, K1, pCa, Kr, Ks

*Note that the listing starts from the least to the most influential ion channel*.

**Figure 4:**
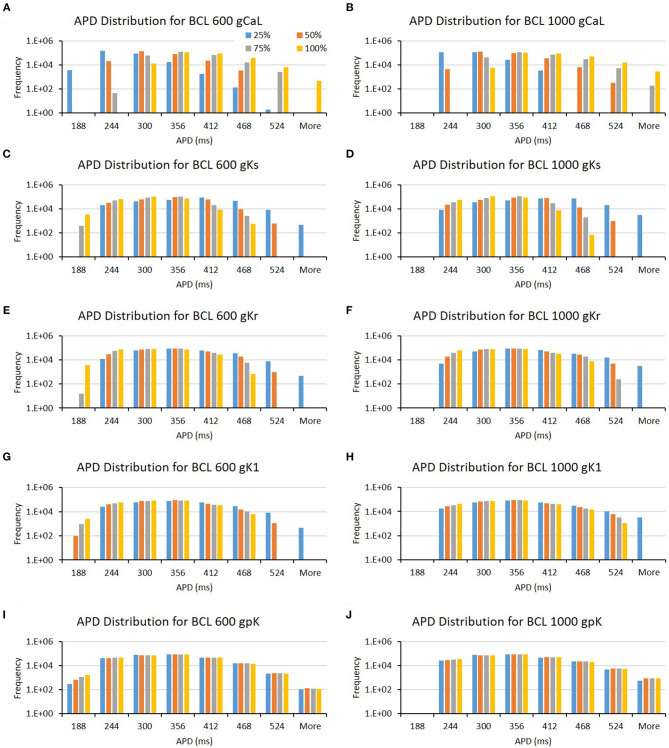
The bar charts show the influence of major ion channels (based on Table 1) for prolonging APD [for BCL 600 ms **(A,C,E,G,I)** and for BCL 1,000 ms **(B,D,F,H,J)**]. The x-axis is the APD groups in milliseconds, and the y-axis is the frequency (number of appearance of APD groups) in logarithmic scale. Four conductance variations are applied (25–100%).

From Table 1, we can observe that the CaL and Ks channels, followed by the Kr channel, play a significant role in prolonging APD. Both BCL 600 and 1,000 ms showed the same results for the three channels. However, even though not for all BCL cases, other channels, such as K1 and pK channels, affect APD prominently only on specific BCL. K1 mostly influences BCL 600 ms, while pK on BCL 1,000 ms. In addition, we can obtain some additional evidence by looking at the bar chart in Figure 4. In Figures 4A,B, long APD is clearly affected by CaL, where moderate-to-normal variation (75–100%) generates long APD. Conversely, low to moderate variation (25–75%) of both Ks and Kr channels can prolong APD, as shown in Figures 4C–F. In contrast, the conductance reduction of the K1 channel, as shown in Figures 4G,H, can trigger long APD (more than 524 ms), especially at the 25% level for both BCL variations. However, the variation of the pK channel, which is listed as one of the major ion channels for prolonging the APD, does not affect the APD as much as the K1 channel, as shown in Figures 4I,J. The downgrading of the pK channel influences APD prolongation mostly on low APD (<188 ms) under BCL 600 ms.

**Figure 5:**
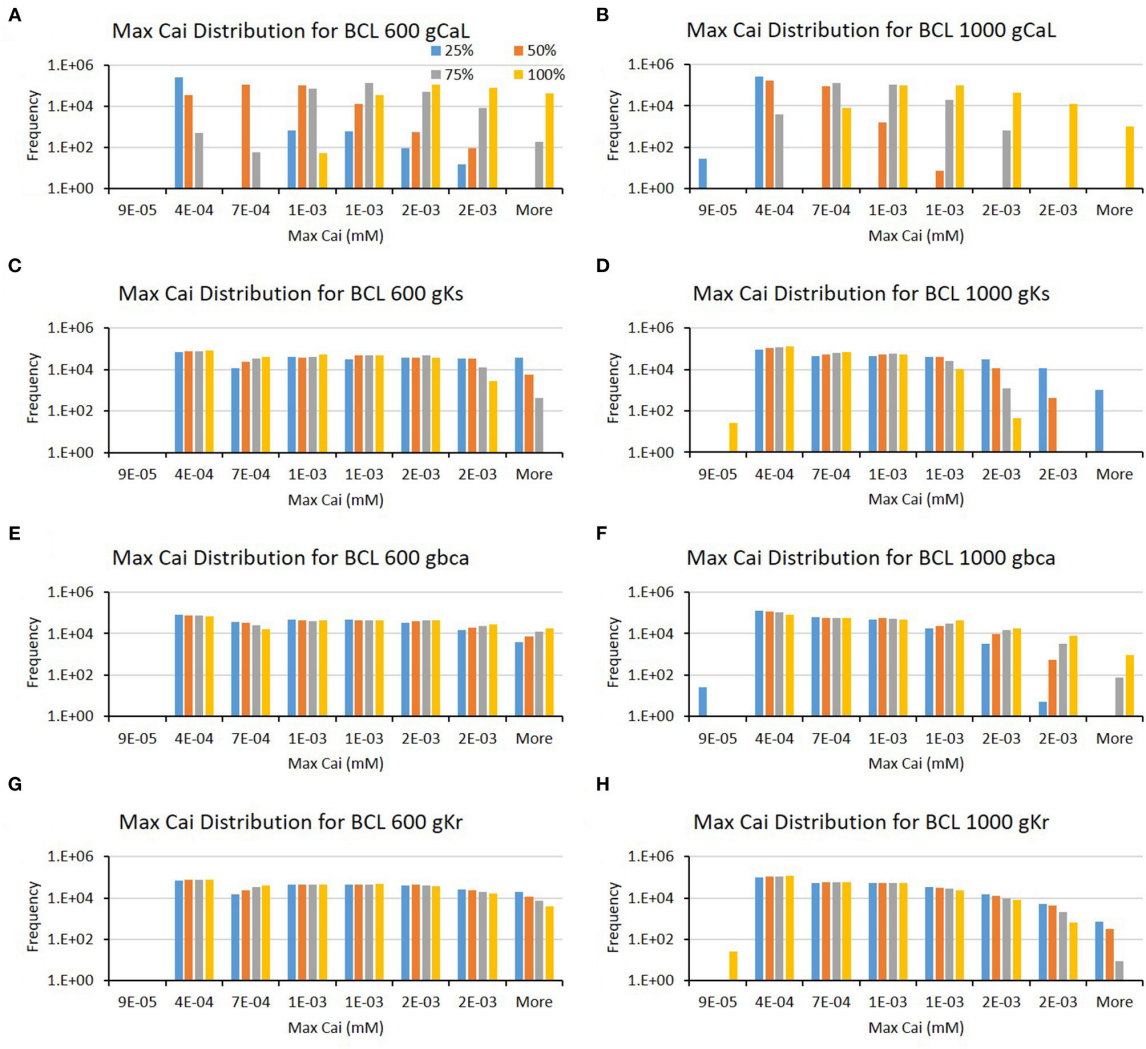
The bar charts show the influence of major ion channels (based on Table 1) for increasing the max Cai [for BCL 600 ms **(A,C,E,G)** and for BCL 1,000 ms **(B,D,F,H)**]. The x-axis is the max Cai groups in millimolar, and the y-axis is the frequency (number of appearance of max Cai groups) in logarithmic scale. Four conductance variations are applied (25–100%).

Furthermore, from Table 1, we can also observe that the major ion channels, such as CaL, Ks, background calcium (bCa), and Kr, can affect the max Cai consistently for both BCL 600 ms and 1,000 ms. The bar charts in Figure 5 also show the influence of the four major ion channels to the max Cai concentration. It can be seen that the reduction of CaL (on Figures 5A,B) and Ks channels (on Figures 5C,D) greatly maximizes the Cai. For the CaL channel, its moderate-to-normal (75–100%) variation can yield high max Cai, whereas, for the Ks channel, it is the low-to-moderate (25–75%) variation. Furthermore, in Figures 5E–H, the bCa channel with the moderate-to-normal variation (50%−100%) and the Kr channel with low to moderate (25–75%) variation can yield high-max Cai. However, these two channels affect more strongly only in BCL 1,000 ms than that in BCL 600 ms.

**Figure 6:**
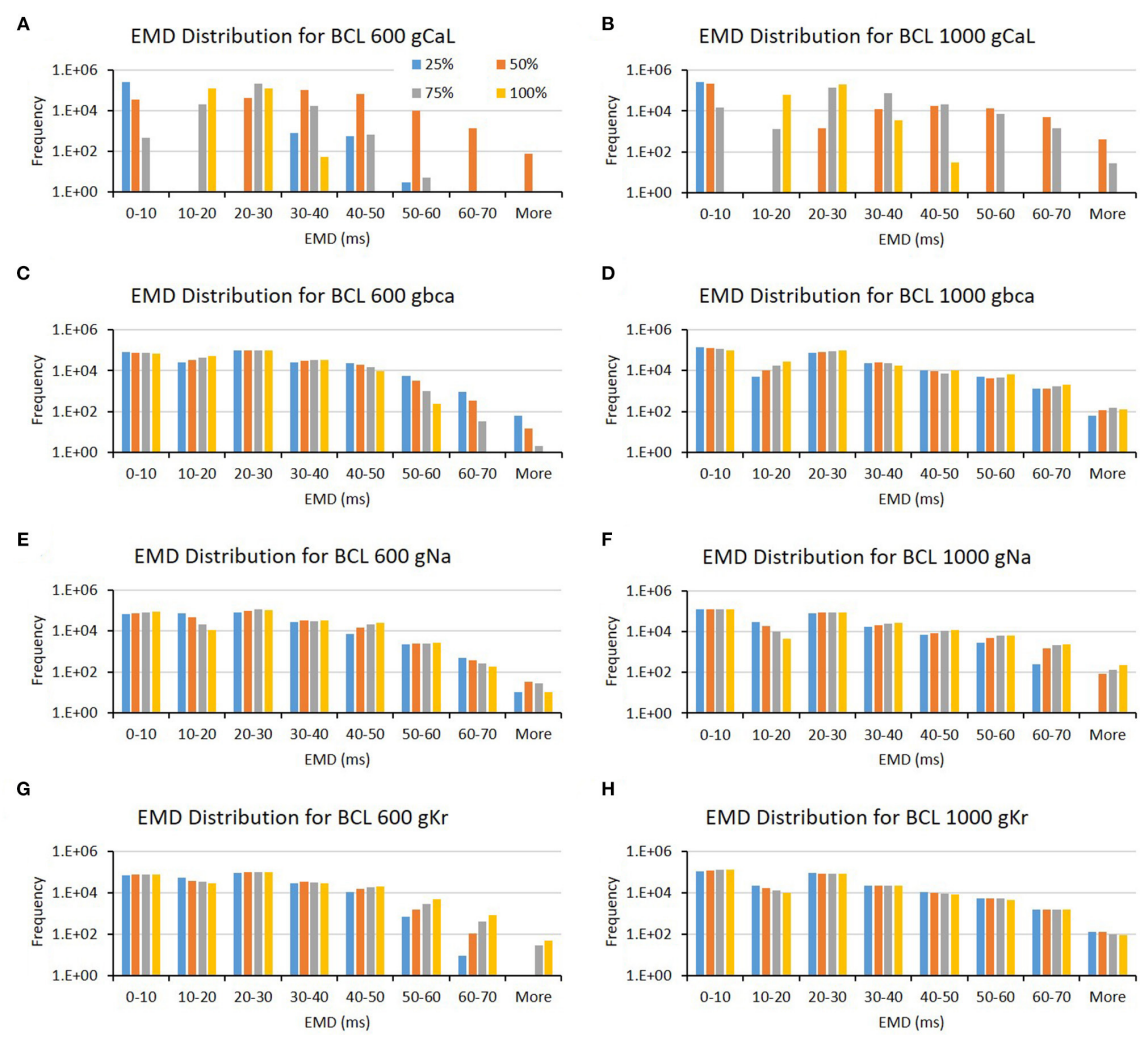
The bar charts show the influence of major ion channels (based on Table 1) for increasing the EMD [for BCL 600 ms **(A,C,E,G)** and for BCL 1,000 ms **(B,D,F,H)**]. The x-axis is the EMD groups in milliseconds, and the y-axis is the frequency (number of appearance of EMD groups) in logarithmic scale. Four conductance variations are applied (25–100%).

The conductance axis configuration for generating EMD prolongation might behave distinctively compared to APD and max Cai, as shown in Table 1. Some major ion channels affecting EMD are CaL and bCa. In addition, the reduction in Na and Kr channels also influences EMD but is more specific for either BCL 600 or BCL 1,000 ms. A more informative description can also be seen in Figure 6. The variation of the CaL channel (Figures 6A,B) at moderate levels (50–75%) significantly influenced the EMD prolongation for both BCL 600 ms and 1,000 ms. In contrast, the reduction of bCa (Figures 6C,D), Na (Figures 6E,F), and Kr (Figures 6G,H) channels lengthen the EMD significantly only in either BCL 600 ms or 1,000, that is, low-to-moderate variation (25–75%) of the bCa channel prolonged EMD in BCL 600 ms, while moderate-to-normal variation (50–100%) of Na and Kr channels prolonged EMD in BCL 1,000 ms.

**Figure 7:**
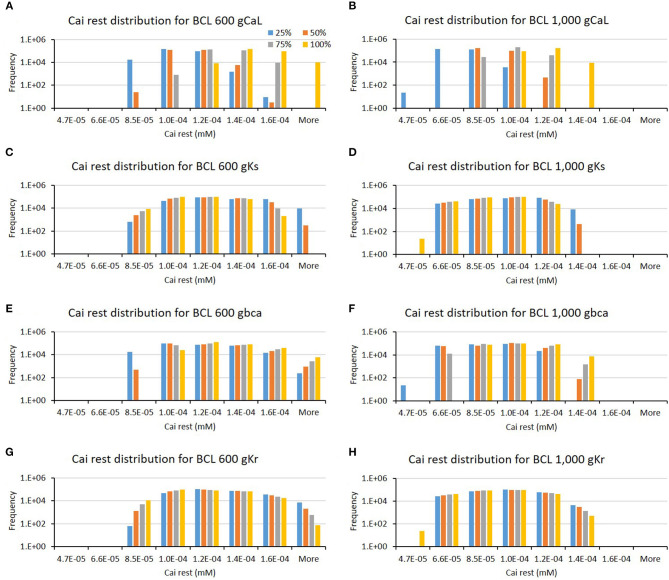
The bar charts show the influence of major ion channels (based on Table 1) on the resting Cai concentration [for BCL 600 ms **(A,C,E,G)** and for BCL 1,000 ms **(B,D,F,H)**]. The **x**-axis is the resting Cai groups in millimolar, and the **y**-axis is the frequency (number of appearance of resting Cai groups) in logarithmic scale. Four conductance variations are applied (25–100%).

**Figure 8:**
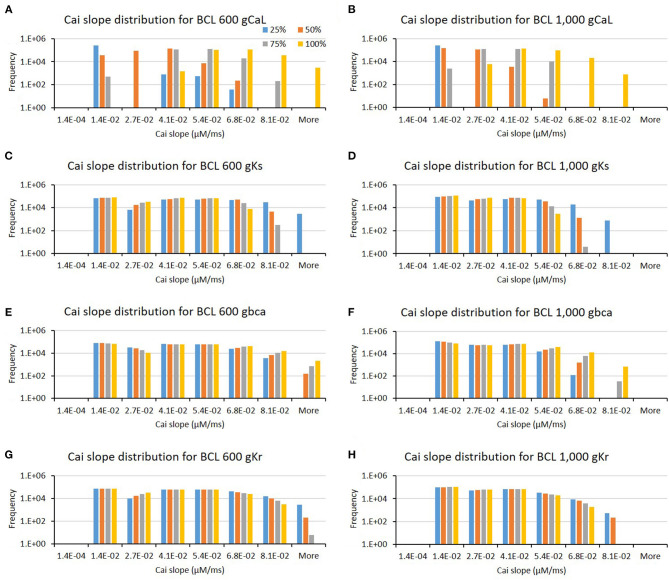
The bar charts show the influence of major ion channels (based on Table 1) for changing the Cai slope [for BCL 600 ms **(A,C,E,G)** and for BCL 1,000 ms **(B,D,F,H)**]. The x-axis is the Cai slope groups in milliseconds, and the y-axis is the frequency (number of appearance of Cai slope groups) in logarithmic scale. Four conductance variations are applied (25–100%).

**Figure 9:**
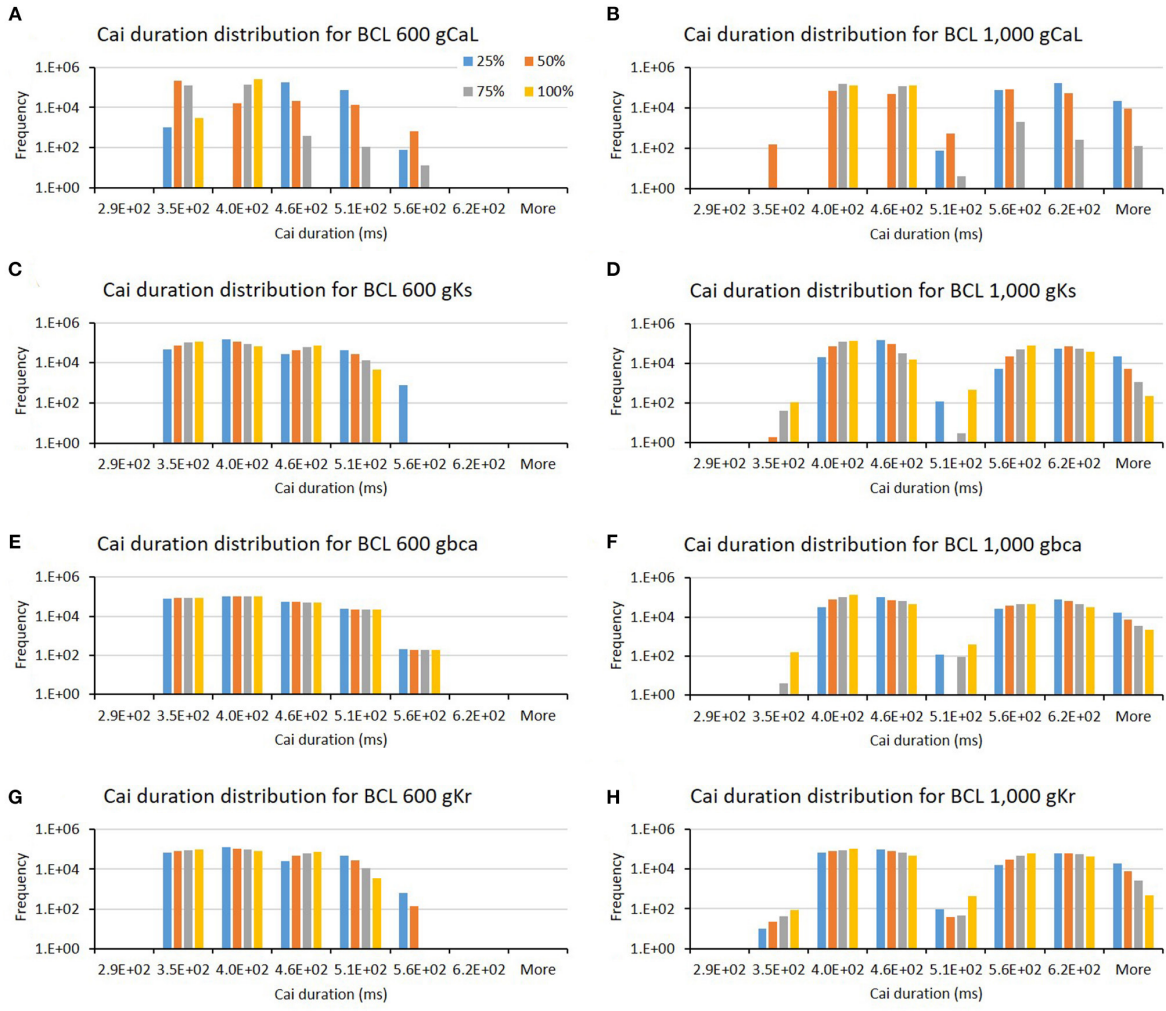
The bar charts show the influence of major ion channels (based on Table 1) for altering the Cai duration [for BCL 600 ms **(A,C,E,G)** and for BCL 1,000 ms **(B,D,F,H)**]. The x-axis is the Cai duration groups in milliseconds, and the y-axis is the frequency (number of appearance of Cai duration groups) in logarithmic scale. Four conductance variations are applied (25–100%).

The influence of four major ion channels on other calcium characteristics, such as resting Cai concentration, Cai slope, and Cai duration, is shown in Figures 7–**9**. Compared to the summary in Table 1, the three calcium attributes show similar configuration of four major ion channels: CaL, Ks, bCa, and Kr channels (except for Cai duration that bCa affects more for BCL 1,000 ms, and pK channels affect dominantly on BCL 600 ms). On Figure 7, moderate-to-normal condition (75–100%) of CaL channel in both BCL 600 and 1,000 ms can significantly yield quite high-Cai rest of more than 0.14 μM. In contrast, low-to-moderate (25–50%) condition of Ks channel in both BCK 600 and 1,000 ms can dominantly generate high-resting Cai of more than 0.14 μM. Similar trends, also, can be observed on Figure 8 where CaL channel with a moderate-to-normal level (75–100%) in both BCL 600 and 1,000 ms can produce a quite high-Cai slope of more than 0.081 μM/ms, in contrary with the influence of low to moderate condition (25–50%) of Ks channel. However, from Figure 9, the CaL channel with low-to-moderate condition (25–50%) can produce quite long Cai duration. For CL 600 ms, the longest Cai duration is observed to be around 560 ms, while, for BCL 1,000, it is more than 620 ms.

**Figure 10:**
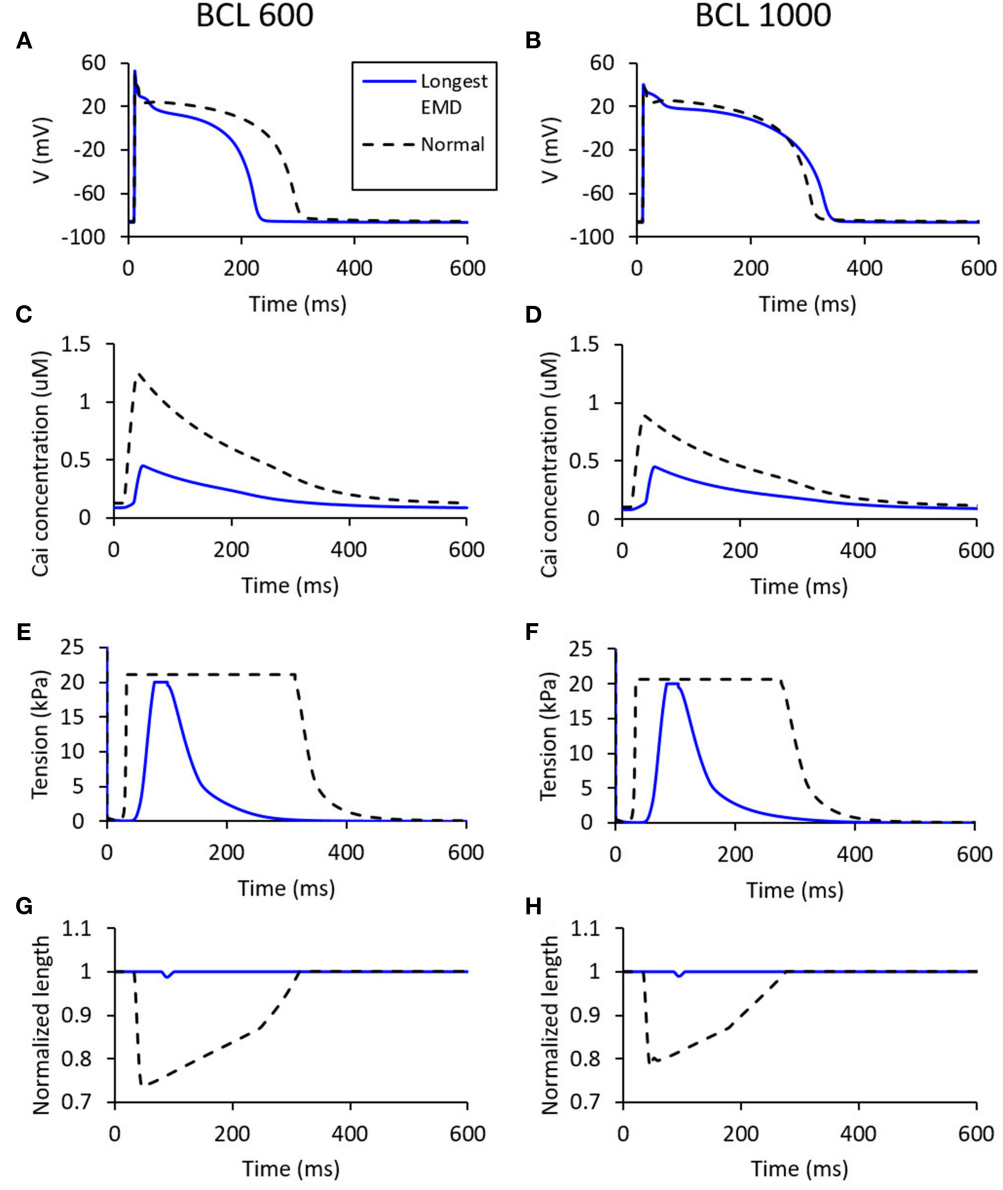
The states for the longest EMD on each BCL variation [for BCL 600 ms **(A,C,E,G)** and for BCL 1,000 ms **(B,D,F,H)**]. The blue line represents the states for a case with the longest EMD, while the dashed black line is the normal case with all channels set to 100% maximum conductance.

Figure 10 shows the states of the cell with the longest EMD (depicted as blue lines), which is represented in the form of membrane potential, tension, normalized length, and Cai concentration of the cell. The longest EMD for BCL 600 ms has a maximum conductance configuration for each channel, such as 100% Ks, 100% Kr, 100% K1, 100% Na, 75% bNa, 50% CaL, 25% bCa, 100 to 100% pCa, and 100% pK. Meanwhile, BCL 1,000 ms has a configuration of 50% Ks, 25% Kr, 100% K1, 100% Na, 100% bNa, 50% CaL, 25% bCa, 50 to 75% pCa, and 100% pK. From Figures 10A,B, we can observe that the longest EMD case for BCL 600 ms has a considerable APD difference compared with the normal case (roughly 75-ms difference), while, for BCL 1,000 ms, the APD difference is quite insignificant. Figures 10C,D show that the longest EMD case has a max Cai concentration <0.5 μM for both BCL variations. Figures 10E,H represent the mechanical contraction of the cell at which both BCL variations show a similar pattern in driving the longest EMD. In Figures 10E,F, we can observe that the tension is cut at 20 kPa because we applied 20 kPa as the “weight” criteria for the cell to start contracting. In Figures 10G,H, length contraction arises once the 20-kPa requirement is achieved.

## Discussion

From the result of the APD matrix map in Figure 3, it is shown that the higher the maximum conductance of the CaL channel, the longer the APD. This is logical because the *I*_*CaL*_ contributes to prolongation during the plateau state of the action potential. Higher maximum conductance means that more calcium ions flow into the cell, causing a longer plateau state that extends the overall APD. Meanwhile, the reduction of Ks channel inversely affects APD prolongation; the lower its conductance, the larger the APD prolongation. The Kr channel also inversely affects APD prolongation, similar to the Ks channel, as shown in Figure 4. These results are consistent with a recent report by Devenyi et al. (2017), who performed population-based sensitivity analysis as proposed by Sobie (2009) by randomly assigning conductance perturbation to the ion channels. They found that *I*_*CaL*_ and *I*_*Ks*_ greatly influence APD, followed by *I*_*Kr*_. Their results also showed a similar pattern on APD prolongation to ours where the higher the maximum *I*_*CaL*_ current, the longer the APD, and, in contrary, the higher the maximum *I*_*Ks*_ and *I*_*Kr*_ current, the shorter the APD. Moreover, a study by Jing et al. (2014) that focuses on the effect of *I*_*Ks*_ current to repolarization dynamics in swine ventricle also showed that the reduction of *I*_*Ks*_ current can prolong the baseline of APDs and increases measures of hysteresis in restitution memory as well as steeper restitution curves.

Furthermore, as a major ion channel for prolonging APD, especially for BCL 600 ms (Table 1), the low-conductance (25%) variation of the K1 channel (as shown in Figure 4) could yield a long APD of more than 524 ms. This result shows that the K1 channel also plays a significant role in APD prolongation, next to CaL, Ks, and Kr. The role of the three aforementioned potassium channels in giving a limited level of redundancy to the cardiac electrical signaling has been recognized as reported by Schmitt et al. (2014) and by Roden (1998) that emphasizes the “repolarization reserve” that describes the overlapping impact of *I*_*Kr*_, *I*_*Ks*_, and *I*_*K*1_ on the repolarization of the action potential. Conversely, the downgrading of the pK channel from both the bar chart in Figure 4 and the APD map in Figure 3 does not strongly influence the prolongation of the APD. These results are consistent with a previous study reported by Devenyi et al. (2017) that the change of *I*_*pK*_ current shows very small effects on APD prolongation indicated by a small APD sensitivity coefficient.

The comparison of the optimum maps in Figure 3 shows that BCL variation clearly affects the map of max Cai and EMD. BCL 600 ms can yield more cases with high max Cai, which is indicated by more yellow to red dots compared to the results from BCL 1,000 ms. This difference in the max Cai map results in distinctive EMD maps for each BCL on Figures 3E,F. As we can clearly observe, BCL 1,000 ms has more no-contraction cases, as indicated by the wider bold blue region in Figure 3F, than BCL 600 ms in Figure 3E. No contraction means that no length shortening occurs in the cell because the tension generated by crossbridge formation is less than the threshold value of 20 kPa, owing to the lack of Cai concentration. Furthermore, the CICR process shows that calcium entering the CaL channel triggers ryanodine receptors to release more calcium from the SR (Endo et al., 1970), which then activates the MF to generate force. The well-known steady-state force-pCa (calcium concentration) curves indicate that the MF calcium cooperativity is described as the Hill coefficient, which can be as high as 5–9 for cardiac muscle (Pfeiffer et al., 2014). High cooperativity means that the availability of calcium is crucial to generating tension in the muscle. This is why no-contraction cases mostly emerge within a low conductance condition (25–50%) of the CaL channel, as shown in Figures 3E,F, where a low Cai concentration is expected to occur.

Furthermore, even though the membrane potential profile of the longest EMD cases for two BCLs has a quite distinctive electrical profile in terms of APD on Figures 10A,B, they inherit similar mechanical responses, as depicted in Figures 10C–H. Compared with the normal case, the longest EMD case tends to have only minor responses, such as a short high-mechanical tension period (Figures 10E,F) and short-length contraction (Figures 10G,H). This is due to the low-max Cai concentration depicted in Figures 10C,D, which have a smaller value compared with the normal case configuration. In other words, the mechanical response for the longest EMD is insignificant compared with the normal case. This result is consistent with the experimental study for isolated myocytes reported by Perreault et al. (1992). Furthermore, a computational study of dyssynchronous canine heart reported by Constantino et al. (2013) emphasized that the deranged Cai handling slows down myofiber-shortening velocity and lowering both myofiber shortening and the stretch rate at the late-activated lateral wall. This causes a delay in the onset of myofiber shortening, therefore rising the EMD prolongation in heart failure.

In addition to the mechanical response of the cases with the longest EMD, it is interesting to note that the effect of conductance variation of some major ion channels that have been discussed might not be able to influence the EMD prolongation. APD prolongation is significantly affected by CaL, Ks, Kr, and K1 channels, while high-max Cai is generated mostly by CaL, Ks, bCa, and Kr channels. In contrast, EMD is greatly affected by the variation in the CaL channel only. From the action potential plot in Figures 2A,B, 10A,B, we can observe that the time for the onset of the action potential is almost similar for all conductance variations. This indicates that the major cause of EMD prolongation is solely the difference in the start of mechanical contraction of the cell, as depicted in the bottom right panel of Figure 1.

Therefore, even though some of the ion channel variations can prolong APD, they might not be able to prolong EMD as they cannot directly influence or trigger the mechanical contraction of the cell. Hence, the CaL channel, as one of the most influential channels for triggering calcium release from the SR, plays a significant role in determining how the cell tension is generated, and, thus, it considerably affects the max Cai and others calcium characteristics, such as resting Cai, Cai slope, and Cai duration in the cell and EMD prolongation. Previous studies by Constantino et al. (2013) and Winslow et al. (1999) also showed similar results in that the abnormality of handling Cai lengthens EMD in DHF.

## Conclusion

In this study, we assessed ion channel sensitivity by varying maximum conductance and BCL of the ion channels to determine the dynamics and interplay among those channels to trigger EMD prolongation. We also examined two quantities that are closely related to EMD: APD and calcium attributes such as max-Cai concentration, resting Cai, Cai slope, and Cai duration. We found that major ion channels, such as CaL, Ks, Kr, and K1, can significantly influence APD prolongation. Furthermore, the calcium characteristics, such as max Cai concentration, Cai rest, Cai duration, and Cai slope, were mostly affected by the variation in the CaL, Ks, bCa, and Kr channels (exception for Cai duration that pK and bCa channels have different degrees of influence for different BCLs). However, among all the aforementioned major ion channels, only the CaL channel can play an important role in EMD prolongation.

In terms of the electrical activity of the cell, we found that all possible cases show a similar time of the onset of the action potential despite the variation in the maximum conductance of ion channels applied on it. As EMD prolongation is measured between the onset of action potential and the start of length shortening, a similar time of firing of the action potential strongly indicates that EMD prolongation depends solely on the mechanical response of the cell. In this regard, the presence of Cai plays a dominant role in giving rise to the mechanical contraction of the cell and is significantly influenced by the variation in the maximum CaL channel conductance. However, there is a restriction that long EMD (>70 ms) can only be generated within 50–75% of the maximum CaL conductance.

Furthermore, ventricular cells with long EMD have shown to inherit insignificant mechanical responses (in terms of how strong the tension can grow and how far length shortening can go) compared with normal cells. Therefore, in a real situation, heart failure that incorporates long EMD is highly likely to result in compromised pump function.

## Data Availability Statement

The original contributions presented in the study are included in the article/supplementary material, further inquiries can be directed to the corresponding author/s.

## Author Contributions

This study is an intellectual product of the entire team. All authors contributed (to varying degrees) to the analyses performed, developing the research concept, designing the simulation, developing the simulation source code, performing the simulation, and writing the manuscript.

## Conflict of Interest

The authors declare that the research was conducted in the absence of any commercial or financial relationships that could be construed as a potential conflict of interest.

## Publisher's Note

All claims expressed in this article are solely those of the authors and do not necessarily represent those of their affiliated organizations, or those of the publisher, the editors and the reviewers. Any product that may be evaluated in this article, or claim that may be made by its manufacturer, is not guaranteed or endorsed by the publisher.
